# Multidimensional structure of the Groningen Frailty Indicator in community-dwelling older people

**DOI:** 10.1186/1471-2318-13-86

**Published:** 2013-08-22

**Authors:** Annemiek Bielderman, Cees P van der Schans, Marie-Rose J van Lieshout, Mathieu HG de Greef, Froukje Boersma, Wim P Krijnen, Nardi Steverink

**Affiliations:** 1 Research and Innovation Group in Health Care and Nursing, Hanze University of Applied Sciences, PO Box 3109, 9701 DC, Groningen, The Netherlands; 2 Practice for Physical Therapy Weustink, Klooster Leuterstraat 24, 3961 AZ, Wijk bij Duurstede, The Netherlands; 3 Julius Center for Health Sciences and Primary Care, University Medical Center Utrecht, PO Box 85500, 3508 GA, Utrecht, The Netherlands; 4 Institute of Human Movement Sciences, University of Groningen, PO Box 1969, 9700 AD, Groningen, The Netherlands; 5 Department of General Practice, Elderly Care Section University of Groningen, University Medical Center Groningen, PO Box 1969, 9700 AD, Groningen, The Netherlands; 6 Department of Health Sciences, Health Psychology Section, University Medical Center Groningen, University of Groningen, PO Box 1969, 9700 AD, Groningen, The Netherlands; 7 Department of Sociology, University of Groningen, PO Box 1969, 9700 AD, Groningen, The Netherlands

**Keywords:** Frailty, Older adults, Screening, Measurement

## Abstract

**Background:**

Due to the rapidly increasing number of older people worldwide, the prevalence of frailty among older adults is expected to escalate in coming decades. It is crucial to recognize early onset symptoms to initiate specific preventive care. Therefore, early detection of frailty with appropriate screening instruments is needed. The aim of this study was to evaluate the underlying dimensionality of the Groningen Frailty Indicator (GFI), a widely used self-report screening instrument for identifying frail older adults. In addition, criterion validity of GFI subscales was examined and composition of GFI scores was evaluated.

**Methods:**

A cross-sectional study design was used to evaluate the structural validity, internal consistency and criterion validity of the GFI questionnaire in older adults aged 65 years and older. All subjects completed the GFI questionnaire (n = 1508). To assess criterion validity, a smaller sample of 119 older adults completed additional questionnaires: De Jong Gierveld Loneliness Scale, Hospital Anxiety Depression Scale, RAND-36 physical functioning, and perceived general health item of the EuroQol-5D. Exploratory factor analysis and Mokken scale analysis were used to evaluate the structural validity of the GFI. A Venn diagram was constructed to show the composition of GFI subscale scores for frail subjects.

**Results:**

The factor structure of the GFI supported a three-dimensional structure of the scale. The subscales Daily Activities and Psychosocial Functioning showed good internal consistency, scalability, and criterion validity (Daily Activities: Cronbach’s α = 0.81, H_s_ = .84, r = −.62; Psychosocial Functioning: Cronbach’s α = 0.80, H_s_ = .35, r = −.48). The subscale Health Problems showed less strong internal consistency but acceptable scalability and criterion validity (Cronbach’s α = .57, H_s_ = .35, r = −.48). The present data suggest that 90% of the frail older adults experience problems in the Psychosocial Functioning domain.

**Conclusions:**

The present findings support a three-dimensional factor structure of the GFI, suggesting that a multidimensional assessment of frailty with the GFI is possible. These GFI subscale scores produce a richer assessment of frailty than with a single overall sum GFI score, and likely their use will contribute to more directed and customized care for older adults.

## Background

Frailty is characterized by a decline in reserve capacity in different domains of functioning, resulting in a decline in mobility, unintended weight loss, an elevated risk of morbidity, an increase in depression and anxiety, institutionalization, and premature death [1,2]. Due to the rapidly increasing number of older people worldwide, the prevalence of frailty among older adults is increasing and expected to escalate in coming decades [3,4]. In order to prevent the detrimental consequences of frailty, like the loss of balance and the decrease in muscle strength and walking speed, it is crucial to recognize early onset symptoms and then initiate appropriate care and specific preventive interventions. A number of review studies have shown that several interventions may be beneficial for older adults in different stages of frailty [5-8].

Early detection of frailty in older adults is feasible with appropriate screening instruments. These screening instruments measure frailty in various ways [9]. Some measurements are based on a clinical assessment by a geriatrician others use performance-based tests or self-report questionnaires. A number of frailty assessment instruments have emerged in the last decade [1,9-23]. These instruments are designed to screen older adults in a valid and feasible way. The majority of these screening instruments include items on physical frailty characteristics like mobility and nutritional status. Only some instruments include items in multiple frailty domains, like the Frailty Index, the Groningen Frailty Indicator, the Tilburg Frailty Indicator and the Edmunton Frail Scale [9]. Especially frailty instruments used for case finding and screening, evaluate frailty dichotomously: persons are considered as either frail or not frail, regardless of the multiple dimensions measured by the instrument [9].

One of these multidimensional screening instruments is the Groningen Frailty Indicator (GFI). The GFI is a widely used screening instrument for identifying frail older adults [22,24]. The GFI consists of 15 self-report items and is a feasible way to assess frailty in both community-dwelling and institutionalized older people [25,26]. Psychometric studies examining the overall internal consistency of the GFI show a range of Cronbach’s α values, from α = 0.68 to α = 0.73, indicating moderate internal consistency [25-27]. Besides feasibility and reliability, the construct and discriminant validity of the GFI were examined in previous research [26].

However, the GFI is being used as a one-dimensional scale based on an overall sum score of 15 items. A person is considered to be frail when the GFI sum score is 4 points or higher [26,27]. The sum score is used as a homogeneous indicator of frailty, without reference to specific problems like sensorimotor functioning, cognitive functioning, mobility, or psychosocial functioning. Consequently, a variety of different frailty-related problems can lead to a sum score of 4 points. We believe that the GFI has the potential to provide more differentiated information about the salience of specific frailty-related problems, and thus direct a more adequately focused program for the care and support frail older adults need. For this reason, an assessment of the various dimensions of frailty is obviously needed.

The main objective of this study was to evaluate the underlying dimensionality of the GFI questionnaire for screening frailty in community-dwelling older persons. In addition, we examined the criterion validity of the GFI subscales. Furthermore, we evaluated the composition of GFI subscale scores for subjects identified as frail based on the currently used cutoff score of 4 points.

## Methods

### Study design

A cross-sectional study design was used to evaluate the structural validity and criterion validity of the GFI questionnaire in older adults aged 65 years and older. In this study, data of older adults living in a small city in a centrally located region of the Netherlands were used (N = 1508). In a smaller sample (N = 119), we examined the criterion validity of the GFI subscales.

### Study sample and data collection

In 2008, 3083 older adults (65 years and older) were approached by their local health authorities to fill in the GFI questionnaire. Besides, a smaller sample of 200 older adults was approached by community centers to fill in the GFI and additional questionnaires. In total, 1508 persons completed the GFI and 119 persons completed the additional questionnaires. Under Dutch legislation, ethical approval was not required in this cross-sectional non-obtrusive observational study. All subjects gave their consent to participate in the study.

### Measures

#### GFI

The GFI is a 15-item screening instrument used to determine the level of frailty [22]. Eight items have two response categories (*yes* / *no*), six items have three response categories (*yes / sometimes / no)*, and one item has a Likert response category (*1*–*10)*. All items were dichotomized to calculate GFI sum scores. A higher GFI sum score indicates a greater level of frailty, with a maximum score of 15. The GFI is displayed in Additional file 1.

To examine criterion validity, we used four additional scales or subscales: De Jong Gierveld Loneliness Scale [28], Hospital Anxiety Depression Scale (HADS) [29], physical functioning subscale of the RAND-36 [30], and the perceived general health item of the EuroQol-5D [31].

#### De Jong Gierveld Loneliness scale

The 6-item De Jong Gierveld scale was used to measure loneliness [28]. This 6-item Likert scale is a reliable and valid instrument for measuring overall, emotional, and social loneliness in large surveys of older adults (Cronbach’s α = 0.61-0.73) [32]. All items have five response categories (*no! / no / more or less / yes / yes!).* After recoding, higher scores indicate greater levels of loneliness.

#### HADS

The Dutch version of the 14-item HADS was used to assess the presence of anxiety and depressive states independent of coexisting general medical conditions [29]. The HADS consists of an anxiety subscale (7 items) and a depression subscale (7 items). In a general population aged 65 years and over, the reliability of both the anxiety and depression subscales as the total scale varied with Cronbach’s α values between 0.71 and 0.8 [29]. Higher scores represent greater anxiety and/or more depressive symptoms.

#### RAND-36

Self-reported physical functioning was assessed using the 10-item physical functioning subscale of the Dutch RAND 36-item Health Survey (RAND-36). The RAND-36 is a reliable and valid scale for measuring different aspects of health in different age groups [30,33]. The overall scale contains eight subscales: physical functioning, social functioning, role limitations caused by physical health problems, role limitations caused by emotional problems, mental health, vitality, bodily pain, and general health perceptions [30]. The physical functioning subscale is a reliable and valid scale for measuring limitations in daily activities due to health problems (Cronbach’s α = 0.92) [30]. The respondent reports to what extent he feels limited in a particular activity (*limited a lot / limited a little / not limited at all).* Raw scores are transformed into index scores ranging from 0 to 100. After transformation, lower scores on the physical functioning subscale indicate more limitations in activities of daily living.

#### EuroQol-5D

Perceived general health was assessed on a Likert scale of 1 to 10, where 10 represents excellent general health. This item represents one item in the overall EuroQol-5D questionnaire [31].

### Statistical analyses

Descriptive statistics were used to report subject characteristics of the study sample.

Structural validity is defined as the degree to which the scores are an adequate reflection of the dimensionality of the construct to be measured [34]. Structural validity was assessed using exploratory factor analysis. Exploratory principal component analysis followed by oblique rotation according to the direct oblimin criterion was conducted to explore factor structure. The number of factors was based on the scree plot evaluation, the size of the eigenvalues, and their confidence intervals. All factors with eigenvalues greater than one were retained. In case an item did not discriminate well between factors, decisions were made based on the content of the item and the results of the reliability analysis of the subscales. Reliability of the factor solution was determined by calculating internal consistency using Cronbach’s α with corresponding 95% Confidence Intervals (CI). A Cronbach’s α coefficient of ≥0.80 was considered “good,” 0.70 – 0.80 “acceptable,” 0.60 – 0.70 “questionable,” 0.50 – 0.60 “poor,” and <0.50 “unacceptable” [35,36].

In addition, scale analysis of the GFI was applied using Mokken item response theory model of monotone homogeneity [37]. Mokken scale analysis tests the homogeneity of the subsets of items of test batteries that are multidimensional by construction [38]. A Loevinger’s scalability coefficient (*H*) of 0.30 - 0.39 indicates a weak scale, *H* 0.40 - 0.49 indicates a moderate scale, and *H* ≥ 0.50 indicates a strong scale [39].

Criterion validity is defined as the degree to which the scores are an adequate reflection of a “gold standard” [34]. To establish criterion validity of the observed GFI subscales, the GFI subscales were compared to related reliable and valid scales considered to be gold standards of the individual dimensions. Positive relations were hypothesized between GFI subscale Psychosocial Functioning and HADS and the Jong Gierveld Loneliness scale. Negative relations were hypothesized between GFI subscale Daily Activities and RAND-36 physical functioning scale, and between GFI subscale Health Problems and Perceived general health (EuroQol-5D). Pearson correlations (two-tailed) between GFI subscales and related scales were calculated. A correlation of <0.30 was considered “low,” 0.30 – 0.60 “moderate,” and > 0.60 “high” [40].

A Venn diagram was constructed to show the composition of GFI subscale scores for all subjects identified as frail based on the currently used cutoff score of 4 points. The diagram provides information about the composition of a score of 4 (or more) points. Only subjects that perceived problems in 25% of the items of each subscale are represented in the Venn diagram. Differences between the groups within the Venn diagram were tested by using the Chi^2^ test for categorical data and ANOVA test for continuous data.

For frail older adults, frequency distributions for different age groups were calculated and tested for dependencies by using the Chi^2^ test and estimation of a log-linear model. We used the factors indicating age (in categories) and perceived problems in the subscales Daily Activities, Psychosocial Functioning, and Health Problems (score on 25% of the subscale items). To increase power, we treated the latter variables as ordinals.

Data from subjects were excluded from further analyses when more than five items (30%) of the GFI were missing. In total, 17 persons were excluded from further analyses because of missing data on the GFI. In the analyzed sample, 1277 persons had no missing data at all, 194 persons had one missing value, 27 persons had two missing values, 4 persons had three missing values and 6 persons had four or five missing values on the GFI. These remaining missing values were imputed by the logistic regression data imputation method [41].

Data were processed using the statistical software SPSS statistics 19 (SPSS Inc., Chicago, IL, USA) and the R statistical programming system (R Development Core Team, 2011). Statistical significance level was set to *p* = 0.05.

## Results

### Participants

A total of 1508 persons participated in the study. Age of the respondents ranged from 65 to 97 years, with a mean (SD) age of 75 (7) years; 49.3% were female, and 41.7% were living alone. Table 1 shows the characteristics of all participants.

**Table 1:** Characteristics of the participants (n = 1508)

	**Overall sample (n = 1508)**	**Main sample (n = 1389)**	**Smaller sample (n = 119)**	**t (df) **^**† **^**or Chi**^**2 **^**(df) **^**‡**^	**p**
**Mean age (y) ± SD**	74.5 ± 6.9	74.3 ± 6.8	77.1 ± 7.7	−3.94 (135.5) ^**†**^	<0.001*
**Age groups, n (%)**					
65 – 69 y	418 (29.2)	392 (29.8)	26 (21.8)	20.01 (4) ^**‡**^	<0.001*
70 – 74 y	363 (25.3)	344 (26.2)	19 (16)		
75 – 79 y	301 (21.0)	274 (20.9)	27 (22.7)		
80 – 84 y	206 (14.4)	181 (13.8)	25 (21)		
≥ 85 y	145 (10.1)	123 (9.4)	22 (18.5)		
**Gender, n (%)**					
Male	730 (50.7)	695 (52.7)	35 (29.4)	30.81 (2) ^**‡**^	<0.001*
Female	709 (49.3)	625 (47.3)	84 (70.6)		
**Educational level, n (%)**					
Low	644 (47.1)	582 (46.4)	62 (55.4)	5.47 (2) ^**‡**^	0.065
Middle	507 (37.1)	467 (37.2)	40 (35.7)		
High	216 (15.8)	206 (16.4)	10 (8.9)		
**Living situation, n (%)**					
Living together	848 (58.3)	807 (60.4)	41 (34.7)	29.37 (1) ^**‡**^	<0.001*
Single living	606 (41.7)	529 (39.6)	77 (65.3)		
**GFI, mean** ± **SD**	3.0 ± 3.0	2.9 ± 3.0	3.4 ± 2.7	−1.77 (1506) ^**†**^	0.078

*Abbreviations*: *GFI* Groningen Frailty Indicator.

*Values are percentages unless indicated otherwise.

^**†**^ Independent t-test results.

^‡^ Chi^2^ test results.

*p < 0.05.

As can be seen in Table 1, the smaller sample differed from the main sample in mean age, gender, and living situation. Compared to the main sample, the smaller sample consisted of persons with a higher average age (77 vs 74 years), relatively more females (71% vs 47%) and more single living persons (65% vs 40%). Educational level and GFI total scores of the smaller sample did not differ significantly from the main sample.

### Factor structure of the GFI

Table 2 shows the factor loadings after oblimin rotation and eigenvalues from the principal component analysis. Evaluation of the scree plot and the size of the eigenvalues strongly suggest that the GFI has a three-dimensional structure, explaining 50.6% of the variance. This analysis produced three subscales: (1) Daily Activities (items 1–4), (2) Psychosocial Functioning (items 11–15), and (3) Health Problems (items 5–10).

**Table 2:** **Factor loadings and eigenvalues from the principal component analysis of the GFI scale (*n*** **= 1508)**

	Factor*		
	**Daily activities**	**Psychosocial functioning**	**Health problems**
1. Shopping	**.646**		
2. Walking outdoors	**.848**		
3. Dressing and undressing	**.855**		
4. Going to the toilet	**.848**		
5. Physical fitness	.326	.303	**.252**
6. Vision problems			**.742**
7. Hearing problems			**.737**
8. Unintentional weight loss			**.374**
9. Use of more than three medicines			**.498**
10. Memory complaints			**.339**
11. Experience of emptiness		**.820**	
12. Missing people around		**.803**	
13. Feeling abandoned		**.789**	
14. Feeling sad/dejected		**.708**	
15. Feeling nervous/anxious		**.598**	
*Initial eigenvalues (95% CI)*	4.42 (4.15-4.69)	1.99 (1.85-2.16)	1.18 (1.10-1.29)
*Cumulative variance (%)*	29.45	42.74	50.58

*Abbreviations*: *GFI* Groningen Frailty Indicator, *CI* confidence interval.

*Factor loadings <0.30 are not presented, except for item 5. Bold loadings correspond to the subscales.

The rotated factors did not clearly discriminate item 5 (“*How do you rate your physical fitness?*”). Based on content and reliability analysis, this item was assigned to factor 3 (subscale Health Problems). Cronbach’s alpha decreased (from .81 to .77) when item 5 was assigned to factor 1 (subscale Daily Activities), and increased (from .47 to .57) when item 5 was assigned to factor 3 (subscale Health Problems).

The GFI subscales Daily Activities and Psychosocial Functioning showed good internal consistency, with Cronbach’s α = 0.81 (95% CI = 0.79-0.83) and Cronbach’s α = 0.80 (95% CI = 0.78-0.82), respectively. By contrast, the subscale Health Problems showed a poor internal consistency (Cronbach’s α = 0.57; 95% CI = 0.54-0.61). In all subscales, Cronbach’s α decreased when any of the items were deleted.

### Scale analysis of GFI subscales

Table 3 shows the scaling coefficients (*H*) from the Mokken scale analyses for each of the GFI subscales. The subscales Daily Activities and Psychosocial Functioning were identified as strong scales, with *H*_s_ = 0.84 and *H*_s_ = 0.54, respectively. On the other hand, the subscale Health Problems was identified as a weak scale (*H*_s_ = 0.35).

**Table 3:** **Scaling coefficients from Mokken scale analyses for items of the GFI subscales (*n*** = 1508)*

**Item**	**Daily activities (item 1–4)**	**Health problems (item 5–10)**	**Psychosocial functioning (item 11–15)**
	*H*_i_ (95% CI)	*H*_i_ (95% CI)	*H*_i_ (95% CI)
1	0.89 (0.84-0.95)	0.40 (0.35-0.45)	0.57 (0.54-0.61)
2	0.83 (0.77-0.89)	0.34 (0.28-0.39)	0.56 (0.52-0.61)
3	0.78 (0.71-0.85)	0.28 (0.23-0.33)	0.58 (0.53-0.62)
4	0.83 (0.74-0.91)	0.30 (0.24-0.35)	0.51 (0.47-0.55)
5	-	0.45 (0.39-0.51)	0.47 (0.42-0.51)
6	-	0.29 (0.23-0.35)	-
*H*_s_	0.84 (0.78-0.89)	0.35 (0.31-0.39)	0.54 (0.50-0.57)

*Abbreviations*: *GFI* Groningen Frailty Indicator, *H*_i_ scaling coefficient of item, *H*_s_ scaling coefficient of total subscale, *CI* confidence interval.

*Interpretation Loevinger’s scaling coefficients: *H*_s_ of 0.30 - 0.40 indicates a weak scale; *H*_s_ of 0.40 – 0.50 indicates a moderate scale; *H*_s_ >0.50 indicates a strong scale.

### Criterion validity of GFI subscales

We assessed the criterion validity of GFI subscales by calculating correlation coefficients among the subscales and four related scales (Jong Gierveld Loneliness Scale, HADS, physical functioning subscale of the RAND-36, HADS, and perceived general health item of the EuroQol-5D) (see Table 4). The subscale Daily Activities was strongly correlated with the RAND-36 physical functioning scale (r = −0.62). The subscale Psychosocial Functioning was strongly correlated with the HADS (r = 0.67) and the Jong Gierveld loneliness scale (r = 0.67). The subscale Health Problems was moderately correlated with the general health rating of the EuroQol-5D (r = −0.48). Furthermore, moderate correlations were found between the Health Problems subscale and the RAND-36 physical functioning (r = −0.53), the HADS (r = 0.36), and the Jong Gierveld Loneliness Scale (r = 0.37). The rating of general health was moderately correlated with all three GFI subscales—Daily Activities, Health Problems, Psychosocial Functioning, (r = −0.31, r = −0.48, r = −0.44, respectively).

**Table 4:** **Pearson correlations between the GFI subscales and related scales (*n*** **= 119)**

	**RAND-36 physical functioning**	**Perceived general health (EuroQol-5D) †**	**HADS**	**De Jong Gierveld Loneliness scale**
**GFI subscale:**	*r* (95% CI)	*r* (95% CI)	*r* (95% CI)	*r* (95% CI)
Daily activities	−0.617*	−0.308	0.264	0.003
	**(−0.72- -0.49)**	(−0.46- -0.13)	(0.08-0.43)	(−0.18-0.19)
Health problems	−0.525	−0.480*	0.355	0.367
	(−0.64- -0.38)	**(−0.66- -0.41)**	(0.18-0.51)	(0.20-0.52)
Psychosocial functioning	−0.237	−0.439	0.668*	0.671*
	(−0.40- -0.06)	(−0.58- -0.28)	**(0.55-0.76)**	**(0.59- 0.76)**

*Abbreviations*: *GFI* Groningen Frailty Indicator, *HADS* Hospital Anxiety and Depression Scale, *CI* confidence interval.

*Bold loadings represent related scales.

†Perceived general health item of the EuroQol-5D questionnaire.

### Composition of GFI score for frail subjects

**Figure 1:**
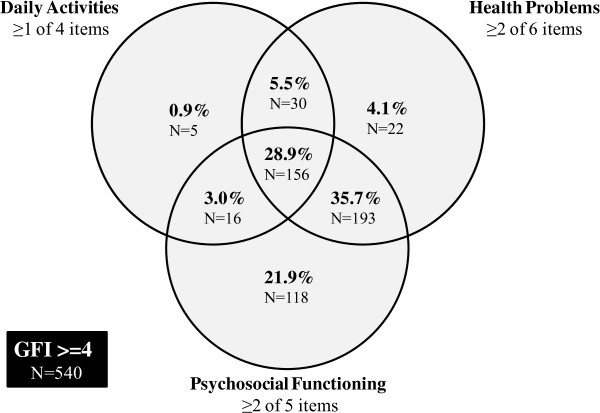
Venn diagram of the frequency distribution of subscale scores for persons with a total GFI-score ≥4 (N = 540).

Figure 1 gives a Venn diagram representation of the distribution of the subscale scores for all subjects with a total GFI score of ≥4 (N = 540). For about one quarter of the frail subjects (26.9%), the GFI score was exclusively composed of perceived problems in one domain. In just a limited number of subjects, the GFI score was exclusively composed of perceived problems in the Daily Activities domain (0.9%) or the Health Problems domain (4.1%). For 21.9% of the frail subjects, the Psychosocial Functioning domain contributed exclusively to the GFI scores.

For almost half of the frail subjects (44.3%), the GFI score was composed of perceived problems in two domains. In only a limited number of subjects, the GFI score was composed of problems in both the Daily Activities and Psychosocial Functioning domains (3.0%), or composed of both the Daily Activities and Health Problems domains (5.5%). For 35.7% of the subjects, both the General Health and the Psychosocial Functioning domain contributed to the GFI scores.

In total, 28.9% of the subjects experienced problems in all three domains of frailty.

The Venn diagram revealed three groups: persons with problems in one subscale (N = 145), those with problems in two subscales (N = 239), and those with problems in all three subscales (N = 156). Table 5 shows the characteristics of these subjects. Subjects that had problems in multiple subscales were significantly older, on average (p < 0.001), and had attained a significantly lower educational level (p = 0.004) than those with problems in only one subscale. Gender, living situation, and financial status did not differ between any of the three groups (p > 0.05).

**Table 5:** Percentages of frail persons (GFI ≥ 4) who experience problems in one, two, or three GFI domains (N = 540)*

	**One domain (N = 145)**	**Two domains (N = 239)**	**Three domains (N = 156)**	**F (df)**^**† **^**or Chi**^**2 **^**(df)**^**‡**^	**p**
**Mean age (y) ± SD**	73.54 ± 5.99	77.46 ± 6.84	80.71 ± 7.19	41.14 (2)^**†**^	<0.001^§^
**Age groups**					
65 – 69 y	27.1	15.2	9.4	76.63 (8)^‡^	<0.001^§^
70 – 74 y	31.4	16.5	10.7		
75 – 79 y	25.0	30.4	20.8		
80 – 84 y	11.4	22.8	26.2		
≥ 85 y	5.0	15.2	32.9		
**Gender**					
Male	41.0	43.3	32.0	5.02 (2)^‡^	0.081
Female	59.0	56.7	68.0		
**Educational level**					
Low	44.6	60.6	65.7	15.26 (4)^‡^	0.004^§^
Middle	40.0	31.7	25.0		
High	15.4	7.7	9.3		
**Living situation**					
Living together	41.0	43.7	43.7	0.30 (2)^‡^	0.861
Single living	59.0	56.3	56.3		
**Financial status**					
No financial problems	83.9	77.2	79.0	2.41 (2)^‡^	0.299
Financial problems	16.1	22.8	21.0		

*Abbreviations: GFI* Groningen Frailty Indicator.

*Values are percentages unless indicated otherwise.

^**†**^ One-way ANOVA test results.

^‡^ Chi^2^ test results.

^§^ p < 0.05.

Among frail subjects, the Chi^2^ test revealed dependency between age and the domains Daily Activities (Chi^2^ = 45.72; df = 4; p < 0.001) and Health Problems (Chi^2^ = 38.69; df = 4; p < 0.001). The data provided no support for an increase of psychosocial problems with increasing age (Chi^2^ = 5.04; df = 4; p = 0.284). ANOVA revealed interactions between age and Health Problems (p < 0.001), and age and Daily Activities (p < 0.001). Age did not interact with Psychosocial Functioning (p = 0.433).

## Discussion

In this study, we examined the structural validity and criterion validity of the GFI questionnaire in older adults. In addition, we evaluated the composition of GFI scores for frail older adults. Our findings support a three-dimensional factor structure of the GFI, in terms of the subscales Daily Activities (items 1–4), Psychosocial Functioning (items 11–15), and Health Problems (items 5–10). This model explains 50.6% of the overall variance. The internal consistency, scalability, and criterion validity of the GFI subscales Daily Activities (Cronbach’s α = .81, H_s_ = .84, r = −.62) and Psychosocial Functioning (Cronbach’s α = .80, H_s_ = .54, r = .67) are good. Consequently, both subscales identify problems in these frailty domains in a reliable and valid way. The internal consistency, scalability, and criterion validity of the GFI subscale Health Problems is less strong (Cronbach’s α = .57, H_s_ = .35, r = −.48). We surmise that the poor reliability and weak scalability of the Health Problems subscale is due to the heterogeneity of items pertaining physical health problems perceived by older adults. The Venn diagram showing the distribution of all subjects with a total GFI score of ≥4 revealed that 27% of older adults had problems in only one domain, 44% had problems in two domains, and 29% had problems in all three domains (see Figure 1). Furthermore, the present data suggest that 90% of the frail older adults experience problems in the Psychosocial Functioning domain.

In the literature, frailty is hypothesized to arise from multiple causes and to affect multiple domains of physical and cognitive functioning [9,42,43]. In different models of frailty, like the Functional Domains model (the accumulation of deficits), the Burden model (the index of health burden) and the Biologic Syndrome model (frailty as a biological syndrome) multidimensional screening instruments are considered to be most appropriate in screening frailty [44]. Although the conceptualization of the multiple domains of frailty is generally used, there is no agreement about the included dimensions in frailty instruments [11,15,45].

In the assessment of frailty, screening instruments are mostly employed in a one-dimensional way. Originally, the GFI applied a cutoff point of a sum score of 4 points or higher, regardless of the number of domains in which an older adult faced problems. In addition, other screening instruments that distinguish different domains, like the Tilburg Frailty Indicator and the Edmunton Frail Scale, also use total sum scores to identify frail older adults [11,46].

We suggest the results of our study may improve the adequacy of screening on frailty and will offer specific indications for intervening in the early onset of frailty. In this study, three separate dimensions of the GFI were established. These results lend support to the use of the GFI screening instrument as a multidimensional tool for the analysis of frailty. When we compare our multidimensional analysis with the originally used one-dimensional approach, as we showed in the Venn diagram, we now get a clearer picture of the underlying problems in the frailty sum scores. Therefore, we question the use of an overall cutoff point to identify frail older adults. It is clinically relevant to use the GFI as a multidimensional scale consisting of three subscales in order to direct the most appropriate care and to provide focused support to older adults facing problems in the different dimensions of frailty. Besides providing support for the use of the GFI screening instrument in a multidimensional way, the present study prompts a fundamental question about using an overall score without delineating specific frailty problems. The question is: Which combinations of pre-conditions are in fact essential for a valid assessment of frailty? The lack of a conceptual model in which frailty is specified results in overestimation and inconsistent identification of frailty in older adults. We propose exploring the possibility of using a conditional cutoff score, one based on *both* the sum score and the subscale scores. We believe this is necessary for establishing a more convergent diagnosis.

We suggest employing a multidimensional assessment of frailty with the GFI, one that uses a conditional cutoff point to establish a more convergent diagnosis of frailty. Because frailty is characterized by a decline in reserve capacity in *different* domains of functioning, we may consider a person to be frail if he or she obtains a GFI sum score of at least 4 points and reports problems in at least two domains of frailty.

A number of relevant methodological issues should be considered in interpreting the results of this study. First, the design was cross-sectional. Thus, we did not evaluate screening results of the GFI over time. Since frailty is a dynamic process that may be reversible, it is relevant to establish the sensitivity of the GFI as a screening instrument [47,48]. So far, the GFI is not been used as an evaluative measurement instrument. Longitudinal studies should clarify the potential of the GFI as an evaluative measurement instrument to assess the changes in frailty status over time.

Second, item 5 of the GFI (“*How do you rate your physical fitness?*”) did not discriminate well among the factors. This finding may be explained by the fact that physical fitness is a multidimensional construct including multiple subcomponents. Furthermore, item 5 is a self-reported measure of physical fitness. It is known that levels of self-reported functioning may be influenced by affective functioning of an older adult [49]. Therefore, the content of item 5 seems to be covered best by the subscale Health Problems, and reliability analysis supports its assignment (higher Cronbach’s α) to this subscale.

Third, a number of relevant personal characteristics were not taken into account in the analyses of our psychometric study. Since our data originated from epidemiological data collected by local health authorities, it contained a limited number of biographic and behavioral data. Therefore, in this study, we could not assess the impact of chronic diseases that may have been present, daily physical activity, physical fitness, and pharmaceutical consumption. It is likely relevant to control for these characteristics to gain more insight into applying the GFI.

## Conclusions

The use of GFI subscale scores is directly relevant to the care of older adults. In our study, we identified three GFI subscales for assessing frailty more specifically. These GFI subscale scores produce a richer assessment of frailty than with the overall sum GFI score, and likely their use will contribute to more directed and customized care for older adults.

## Competing interests

The authors have no conflicts of interest related to the content of this manuscript.

## Authors’ contributions

AB, MdG, FB and NS were involved in the conception and design of the study. MvL performed the data acquisition. AB, MdG, WK and NS were responsible for the data analysis, interpretation of data, and in writing the manuscript. FB and CvdS provided valuable comments during the research process and were involved in revising the manuscript. All authors have read and approved the final manuscript.

## Pre-publication history

The pre-publication history for this paper can be accessed here:

http://www.biomedcentral.com/1471-2318/13/86/prepub

## Supplementary Material

Additional file 1 The Groningen Frailty Indicator - (GFI). Click here for file
