# Supplementary material for: Multidimensional structure of the Groningen Frailty Indicator in community-dwelling older people
Source: BMC Geriatr. 2013 Aug 22;13:86. doi: 10.1186/1471-2318-13-86 (PMC3766248; doi:10.1186/1471-2318-13-86)
Supplement: Additional file 1 — The Groningen Frailty Indicator - (GFI). [file 1471-2318-13-86-S1.pdf]

# Additional file 1: The Groningen Frailty Indicator (GFI)

## The Groningen Frailty Indicator (GFI)

**Daily Activities**

Are you able to carry out these tasks independently without any help? The use of resources such as walking stick, walking frame, wheelchair, is considered independent.

1. Shopping
2. Walking around outside (around the house or to the neighbors)
3. Dressing and undressing
4. Going to the toilet

**Health Problems**

5. How do you rate your physical fitness? (scale 0 to 10)
6. Do you experience problems in daily life due to poor vision?
7. Do you experience problems in daily life due to being hard of hearing?
8. During the last 6 months, did you lost a lot of weight unwillingly? (3 kg in 1 month or 6 kg in 2 months)
9. Do you take 4 or more different types of medicine?
10. Do you have any complaints about your memory?

**Psychosocial Functioning**

11. Do you sometimes experience an emptiness around you?
12. Do you sometimes miss people around you?
13. Do you sometimes feel abandoned?
14. Did you felt downhearted or sad recently?
15. Did you felt nervous or anxious recently?

Scoring:
*Questions 1–4:* Yes = 0; No = 1
*Question 5:* 0–6 = 1; 7–10 = 0
*Questions 6–9:* Yes = 1; No = 0
*Question 10:* Yes = 1; Sometimes = 0; No = 0
*Question 11–15:* Yes = 1; Sometimes = 1; No = 0
